# Relation of Gender to the Occurrence of AKI in STEMI Patients

**DOI:** 10.3390/jcm11216565

**Published:** 2022-11-05

**Authors:** Shir Frydman, Ophir Freund, Ariel Banai, Lior Zornitzki, Shmuel Banai, Yacov Shacham

**Affiliations:** Department of Cardiology, Tel-Aviv Sourasky Medical Center Affiliated to the Sackler Faculty of Medicine, Tel-Aviv University, Tel-Aviv 64239, Israel

**Keywords:** STEMI, AKI, gender, risk, prognosis, mortality

## Abstract

Patients undergoing percutaneous coronary interventions (PCIs) are prone to a wide range of complications; one complication that is constantly correlated with a worse prognosis is acute kidney injury (AKI). Gender as an independent risk factor for said complications has raised some interest; however, studies have shown conflicting results so far. We aimed to investigate the possible relation of gender to the occurrence of AKI in STEMI patients undergoing PCI. This retrospective observational study cohort included 2967 consecutive patients admitted with STEMI between the years 2008 and 2019. Their renal outcomes were assessed according to KDIGO criteria (AKI serum creatinine ≥ 0.3 mg/dL from baseline within 48 h from admission), and in-hospital complications and mortality were reviewed. Our main results show that female patients were older (69 vs. 60, *p* < 0.001) and had higher rates of diabetes (29.2% vs. 23%, *p* < 0.001), hypertension (62.9% vs. 41.3%, *p* < 0.001), and chronic kidney disease (26.7% vs. 19.3%, *p* < 0.001). Females also had a higher rate of AKI (12.7% vs. 7.8%, *p* < 0.001), and among patients with AKI, severe AKI was also more prevalent in females (26.1% vs. 14.5%, *p* = 0.03). However, in multivariate analyses, after adjusting for the baseline characteristics above, the female gender was a non-significant predictor for AKI (adjusted OR 1.01, 95% CI 0.73–1.4, *p* = 0.94) or severe AKI (adjusted OR 1.65, 95% CI 0.80–1.65, *p* = 0.18). In conclusion, while females had higher rates of AKI and severe AKI, gender was not independently associated with AKI after adjusting for other confounding variables. Other comorbidities that are more prevalent in females can account for the difference in AKI between genders.

## 1. Introduction

Patients undergoing percutaneous coronary interventions (PCIs) are prone to a wide range of complications, with numerous investigations performed to predict the subset of patients that are at greater risk [1,2,3,4]. Gender as an independent risk factor has raised some interest, with early reports suggesting females have a greater risk of mortality or adverse events [5,6,7], while more recent reports regarding patients undergoing primary percutaneous intervention (PPCI) have demonstrated conflicting results and attributed gender differences in prognosis to other risk factors [8,9,10,11,12,13]. Among the common and serious complications of acute coronary syndrome (ACS), in general, and ST elevation myocardial infarction (STEMI), in particular, is acute kidney injury (AKI), with numerous studies describing an association of AKI with higher rates of morbidity and mortality [14,15,16,17]. Previous studies have analyzed multiple risk factors for AKI in STEMI patients, including hemodynamic state, the use of contrast materials, hypoxia, inflammation, age, and prior medical conditions; however, gender-related stratification is scarcely found [18,19,20]. Several studies have tried to evaluate gender differences in the intensive care setting and have found that although AKI was more common in males, this difference was diminished in older subsets of patients [21,22]. Our study aimed to investigate the possible relation of gender to the occurrence of AKI in STEMI patients undergoing PCI.

## 2. Methods

### 2.1. Study Population

This is an observational study that was performed in a single tertiary referral hospital (Tel-Aviv Sourasky Medical Center) with a 24/7 PPCI. We evaluated 2967 consecutive patients that were admitted to the cardiac intensive care unit (CICU) with a diagnosis of acute STEMI between January 2008 and December 2019. From our initial cohort, patients were excluded if records of their renal function were missing (*n* = 18) or if they required chronic peritoneal dialysis or hemodialysis (*n* = 5). 

The diagnosis of STEMI was made if a typical history of chest pain occurred with diagnostic electrocardiographic changes and serial elevation of serum cardiac biomarkers [23]. The diagnosis was verified for each patient before inclusion in the cohort. PPCI was carried out in patients with symptoms of under 12 h in duration and in patients with symptoms of 12–24 h in duration if pain began upon admission. Treatments with statins, renin/angiotensin blockers, and β-blockers were started in all patients unless contraindicated. Following PCI normal saline infusion was given for 12 h at a rate of 1 mL/kg/h or lower if patients had overt heart failure. 

We retrieved the baseline demographics and medical history, treatment characteristics, and laboratory results of all included patients (*n* = 2944). The left ventricular ejection fraction was measured within the first 48 h using bedside echocardiography for all patients. In-hospital mortality and complications occurring during hospitalization were evaluated for all patients. Complications included acute renal failure, cardiogenic shock, the need for intra-aortic balloon pump (IABP), new-onset ventricular tachycardia (VT)/fibrillation (VF) episodes, bleeding (requiring blood transfusion), and stent thrombosis.

The study was conducted according to the Declaration of Helsinki and approved by the Tel-Aviv Sourasky Medical Center review board (TLV-16-0224). Informed consent was obtained from all subjects involved in the study.

### 2.2. AKI-Related Definitions

The serum creatinine was regularly evaluated upon patient admission, prior to primary PCI, and at least once daily during hospitalization. The estimated glomerular filtration rate (eGFR) was calculated with the Chronic Kidney Disease Epidemiology Collaboration (CKD-EPI) equation, and a presenting eGFR of under 60 mL/min/1.73 m² was used for chronic kidney disease (CKD) definition [24]. AKI was defined according to the “kidney disease: improving global outcomes” (KDIGO) criteria [25]. Severe AKI was defined as a peak creatinine level of more than double the admission creatinine level, which correlated to stages 2 and 3 of the KDIGO AKI staging [25].

AKI recovery was assessed for each patient and determined by a return to creatinine level within 0.3 mg/dL of that at baseline. Early recovery refers to recovery from AKI within 72 h after AKI diagnosis [26]. Partial recovery refers to an improvement of creatinine to a level higher than 0.3 mg/dL of baseline level within 72 h.

### 2.3. Statistical Analysis

Data were displayed as means (±standard deviation) or medians (25–75%) for normally/non-normally distributed continuous variables. Categorical variables were displayed as numbers (percentages). Chi-square tests were used for the comparison of categorical variables. An independent *t*-test or the Mann–Whitney U test was used to compare normally/non-normally distributed continuous variables. Bivariate logistic regression models were used for multivariate analyses of independent association with AKI (among the entire cohort) and for severe AKI (among patients with AKI), and adjusted odds ratios (AOR) with 95% confidence intervals (CI) were calculated. For both models, variables were selected by the research group after considering both their significance in the univariate analysis and clinical reasoning (the clinical relevance of a variable to both AKI and gender). Gender was obligatory for both models. For all analyses, a two-tailed *p*-value of <0.05 was considered significant. We performed the analyses using the SPSS software version 26 (SPSS Inc., Chicago, IL, USA). 

## 3. Results

### 3.1. Baseline Characteristics and Effect of Gender on AKI

Overall, 2944 patients were included in the study, of whom 2400 (81%) were males, and 544 (19%) were females. The demographic and clinical baseline characteristics are presented in Table 1. Compared with males, females had a higher percentage of AKI, with lower baseline and peak creatinine levels. The estimated GFR levels upon admission were similar between the genders. Females were older and had higher rates of hyperlipidemia, hypertension, diabetes, and CKD. On the other hand, females had lower rates of past myocardial infarctions and smoked less. In addition, females had less severe coronary artery disease. Longer times to the emergency department and to reperfusion were noted for females, while the door-to-balloon time was similar.

Among the patients with AKI (*n* = 255), 45 (18%) patients had severe AKI, 124 (49%) patients had an early recovery in their creatinine, and 52 (20%) had a partial recovery. A comparison of kidney-related outcomes between males and females with AKI is presented in Table 2. As shown, females with AKI had higher rates of severe AKI, while early and partial recovery were similar between the genders.

### 3.2. Gender as an Independently Associated Factor for AKI

To evaluate the independent association of gender with AKI, we created a multivariate regression model, presented in Table 3. When accounting for the main baseline characteristics, the female gender was not found to be independently associated with AKI (AOR 1.01, 95% CI 0.73–1.40, *p* = 0.94). Age (AOR 1.04, 95% CI 1.02–1.05, *p* < 0.001), hypertension (AOR 2.02, 95% CI 1.48–2.75, *p* < 0.001), and CKD (AOR 2.37, 95% CI 1.71–3.30, *p* < 0.001) remained independently associated with AKI.

We performed a similar analysis for severe AKI among the patients with AKI (Table 4). In the multivariate regression model, the female gender was not found to be independently associated with AKI when accounting for the other baseline characteristics (AOR 1.65, 95% CI 0.80–1.65, *p* = 0.18). 

### 3.3. Effect of Gender and AKI on In-Hospital Outcomes

**Figure 1:**
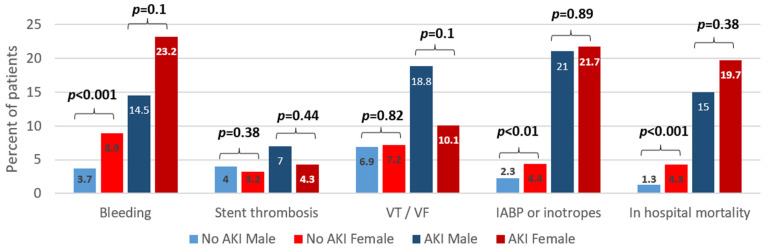
In-hospital adverse outcomes divided by AKI status and compared between genders. VT, ventricular tachycardia; VF, ventricular fibrillation; IABP, intra-aortic balloon pump.

The in-hospital adverse outcomes were divided by the AKI status and are presented in Figure 1; each was compared between the males and females. Among the patients without AKI, females had more bleeding events, had higher rates of cardiogenic shock requiring inotropes or IABP, and had higher in-hospital mortality. Among the patients with AKI, a similar trend was shown for bleeding and in-hospital mortality (higher in females), while it was not statistically significant. Contrary to the patients without AKI, cardiogenic shock had similar rates in the females and males with AKI. The occurrence of VT/VF showed a trend to be higher in males, but only among patients with AKI, although this was not statistically significant.

## 4. Discussion

Our study demonstrated that among patients with STEMI, females have a higher rate of AKI; however, their alleged higher risk for kidney injury, which was initially significant, diminished after correction for background risk factors. The same effect was seen when observing the subgroup of patients with severe AKI. However, we did find females to be at a greater risk for some known complications in comparison to their male counterparts; this was true only for the subset of patients without AKI, as discussed below.

Susceptibility to AKI in males and females was hypothesized to differ due to several mechanisms. Baseline differences in glomerular structure and the effect of sex hormones on renal physiology were possible mechanisms [27,28]. The higher rates of known risk factors for renal injury, such as dyslipidemia, diabetes, and hypertension, in males, were also considered to affect the rate of AKI [29]. In 2012, the KDIGO practice guidelines considered the female sex to be among the shared susceptibility factors that confer a high risk of hospital-acquired AKI [25]. On the contrary, an analysis of 28 studies with over 6.5 million patients revealed that the female gender was associated with protection against hospital-acquired AKI [30]. Our study found that the known risk factors for AKI were more common in females among patients with STEMI. In coherence with those risk factors, AKI was found to occur in a significantly higher proportion of females; however, the tendency to AKI was limited and non-significant in the regression analysis. Schmucker et al. analyzed the occurrence of AKI in 3810 patients with STEMI and did not find a correlation between gender and AKI in accordance with our results [18].

Gender differences in STEMI complications and prognoses have raised some interest recently, with some recently published studies finding females at greater risk of mortality, as well as reduced left ventricular function [11,12]. Furthermore, previous studies have demonstrated that STEMI patients who presented with AKI or developed AKI during their admission were more prone to various complications and mortality; one large-scale meta-analysis by Pickering et al. found AKI to be associated with up to a three-fold increase in mortality [20,31,32]. Our cohort also found that AKI was linked to bleeding, a need for hemodynamic support, and mortality. However, we questioned whether there was a different effect in males and females in this regard, and our results add an interesting aspect of gender distribution. While females were at significantly higher risk of complications in the “no-AKI” subgroup, the relative risk was not found in the AKI group, in which males and females had similar rates of complications. This change might suggest that AKI can be a marker for male patients who have additional comorbidities, and therefore, they are at a greater baseline risk for adverse outcomes.

Mortality in STEMI patients is obviously an important and well-studied field of research, with numerous scores and risk factors linked to greater mortality, including gender, as discussed earlier [33,34,35,36,37]. Previous works have related the higher mortality rates in females with delayed diagnosis and longer door-to-balloon times, as well as added common risk factors [38,39,40,41,42]. It is worth mentioning that we found three factors to be independently linked to AKI, including older age, baseline hypertension, and CKD, all of which are known risk factors for mortality and were more common in the females of our cohort [43,44]. Our results lie in agreement with those articles but highlight the fact that gender itself cannot account for the higher rates of mortality in the presence of AKI as a predisposing background risk.

This study has several limitations. This is a single-center retrospective study, and even with the large size of our cohort, its generalizability to other populations is limited. The study population was non-randomized from a tertiary referral center; hence, a selection bias is possible. We included consecutive patients and performed multivariate analyses to minimize any effect of confounding factors in this regard. Data regarding additional treatments throughout the hospitalization, such as statins or diuretics, were not available for many patients, and their effect between genders could not be assessed. Finally, data regarding patients’ kidney function after hospital discharge were not present, and therefore, we could not account for any long-term improvement or worsening between genders.

In conclusion, our study was set to assess the relation of gender to the occurrence of acute renal injury in STEMI patients. Indeed, females had higher rates of AKI; however, gender was not independently associated with AKI after adjusting for other confounding variables. Some of the baseline characteristics that are more prevalent in females were found to be independently linked to the development of AKI. Therefore, while gender by itself does not predict a predisposition to renal injury, it certainly should be noted.

## Figures and Tables

**Table 1:** Cohort demographic and clinical characteristics, compared by gender ^a^.

Variable	Male (*n* = 2400)	Female (*n* = 544)	Missing Data *n* (%)	*p*
Age	60 (±12)	69 (±13)		<0.001
Hyperlipidemia	1150 (47.9)	312 (57.4)		<0.001
Hypertension	991 (41.3)	342 (62.9)		<0.001
Diabetes	552 (23)	159 (29.2)		<0.001
Past Myocardial infarction	404 (16.8)	61 (11.2)		<0.001
Smoker	1283 (53.3)	201 (36.9)		<0.001
Chronic kidney disease	463 (19.3)	145 (26.7)		<0.001
Ejection fraction (%)	47.4 (±8)	46.4 (±8)		0.02
Time to ER, min	120 (60–360)	180 (75–400)	33 (1.1)	<0.001
Door to balloon, min	45 (30–60)	45 (30–60)	281 (9.5)	0.36
Time to reperfusion, min	165 (105–450)	240 (125–600)	281 (9.5)	<0.001
Coronary artery vessel disease			27 (0.9)	<0.001
1	957 (40.2)	264 (49.3)		
2	760 (31.9)	127 (23.7)		
3	665 (27.9)	144 (26.9)		
Estimated GFR	75.5 ± 20	75.8 ± 24		0.84
Creatinine, admission	1.07 (0.95–1.22)	0.95 (0.82–1.11)		<0.001
Creatinine, peak	1.07 (0.96–1.24)	0.96 (0.82–1.22)		<0.001
Troponin-I, admission	0.91 (0.06–28.4)	1.36 (0.09–19.3)	694 (23.6)	0.21
Troponin-I, peak	47 (7.4–299.6)	30 (6–178.2)	694 (23.6)	<0.01

Values are presented as numbers (%) for categorical variables and as means (±SD) or medians (IQR) for normally/non-normally distributed continuous variables. ^a^ The total number of missing data in the dataset is 2010 (4%).

**Table 2:** Renal outcomes among patients with acute kidney injury, compared by gender.

Variable	Male (*n* = 186)	Female (*n* = 69)	*p*
Acute kidney injury	186 (7.8)	69 (12.7)	<0.001
Baseline Chronic kidney disease	77 (48.7)	39 (65)	0.03
Estimated GFR, admission	58.5 ± 23	57.6 ± 25	0.80
Creatinine, admission	1.24 (1.05–1.55)	1.10 (0.91–1.29)	<0.01
Creatinine, peak	1.81 (1.45–2.49)	1.68 (1.44–2.28)	0.24
Severe AKI ^a^	27 (14.5)	18 (26.1)	0.031
AKI early recovery ^b^	91 (48.9)	33 (47.8)	0.88
AKI partial recovery ^c^	42 (22.6)	10 (14.5)	0.15
AKI any recovery ^d^	133 (71.5)	43 (62.3)	0.16

^a^ Defined as peak creatinine higher than double the admission creatinine level. ^b^ Defined as recovery of creatinine to within 0.3 mg/dL of baseline level within 72 h. ^c^ Defined as improvement of creatinine to more than 0.3 mg/dL of baseline level within 72 h. ^d^ Including both early and partial recovery.

**Table 3:** Multivariate regression model of independent association with AKI.

Variable	AOR	95% CI	*p*
Female	1.01	0.73–1.40	0.94
Age	1.04	1.02–1.05	<0.001
Hypertension	2.02	1.48–2.75	<0.001
Diabetes	1.26	0.94–1.69	0.12
Chronic kidney disease	2.37	1.71–3.30	<0.001

**Table 4:** Multivariate regression model of independent factors for severe AKI among patients with AKI.

Variable	AOR	95% CI	*p*
Female	1.65	0.80–1.65	0.18
Age	1.03	0.99–1.06	0.09
Hypertension	1.13	0.48–2.61	0.78
Diabetes	1.73	0.88–3.38	0.11

## Data Availability

The data presented in this study are available on request from the corresponding author.
